# Supplementary material for: A Splice Variant in SLC16A8 Gene Leads to Lactate Transport Deficit in Human iPS Cell-Derived Retinal Pigment Epithelial Cells
Source: Cells. 2021 Jan 18;10(1):179. doi: 10.3390/cells10010179 (PMC7831140; doi:10.3390/cells10010179)
Supplement: Supplementary file 1 [file cells-10-00179-s001.pdf]

A

Samples	Ratio	Results
#3130-c6n (control)	0.5	< 0.9 myco -
#3130-c7 (control)	0.4	< 0.9 myco -
#4024-c1 (case)	0.3	< 0.9 myco -
#4024-c7 (case)	0.5	< 0.9 myco -
negative control	0.1	< 0.9 myco -
positive control	26.0	> 1.2 myco +

B

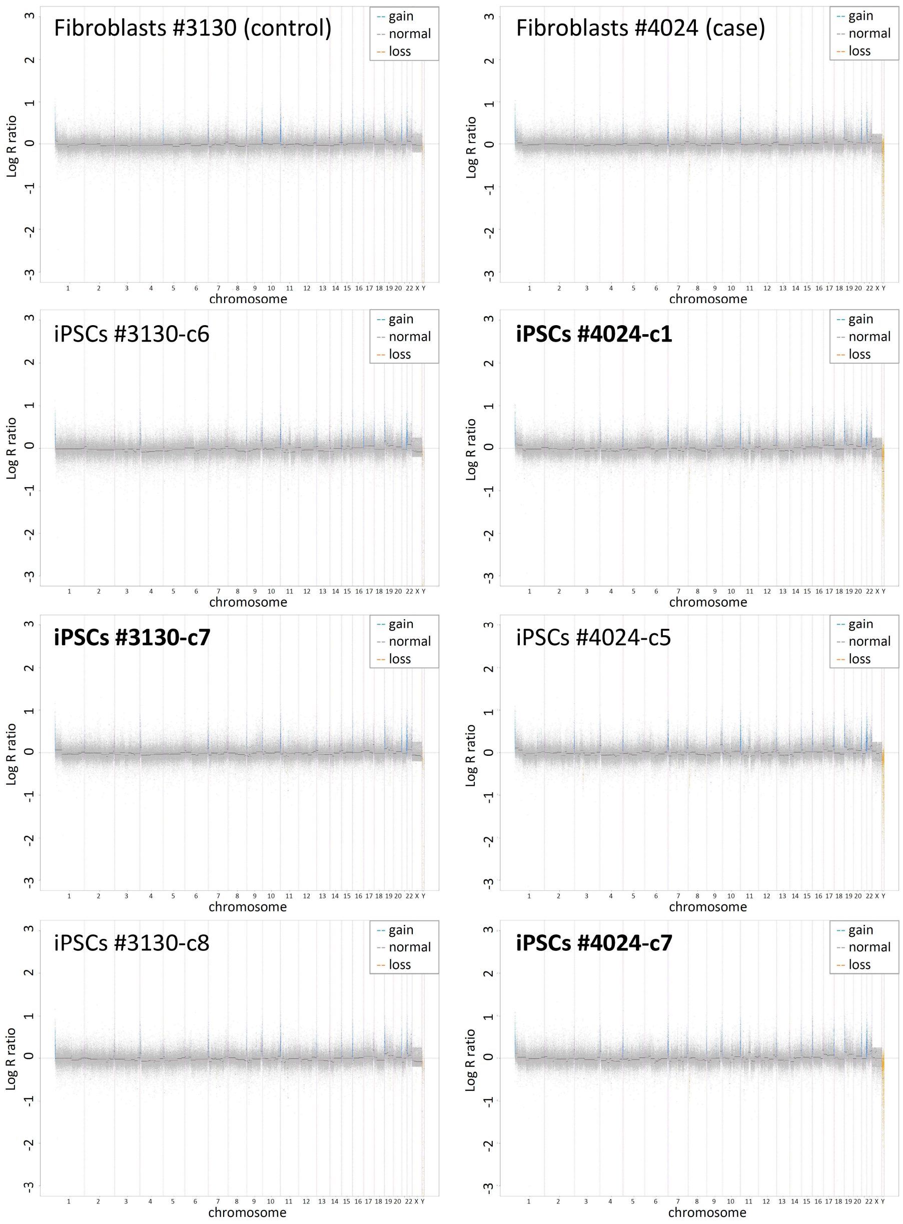

Figure S1. (**A**) Absence of mycoplasma in iPSC lines; (**B**) Copy number variation plot analysis of fibroblasts versus iPSCs. Copy number aberrations are represented in Log R ratio plots as gain (i.e. duplication or amplification) or loss (deletion) in copy number relative to the baseline. Individual Log R ratios in normal regions tend to be near zero. Calculation of Log R ratio is made by comparing experimental data to canonical genotype clusters. iPSC clones used in the experiments are shown in bold.

Slc16a8

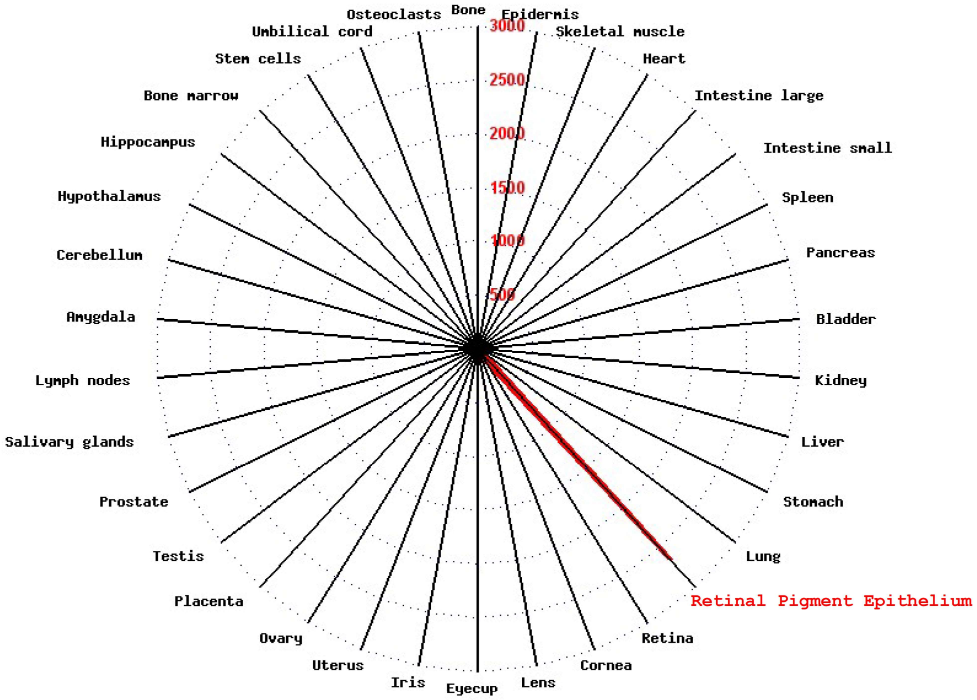

Figure S2. Restricted expression of *Slc16a8* in mouse tissues. .

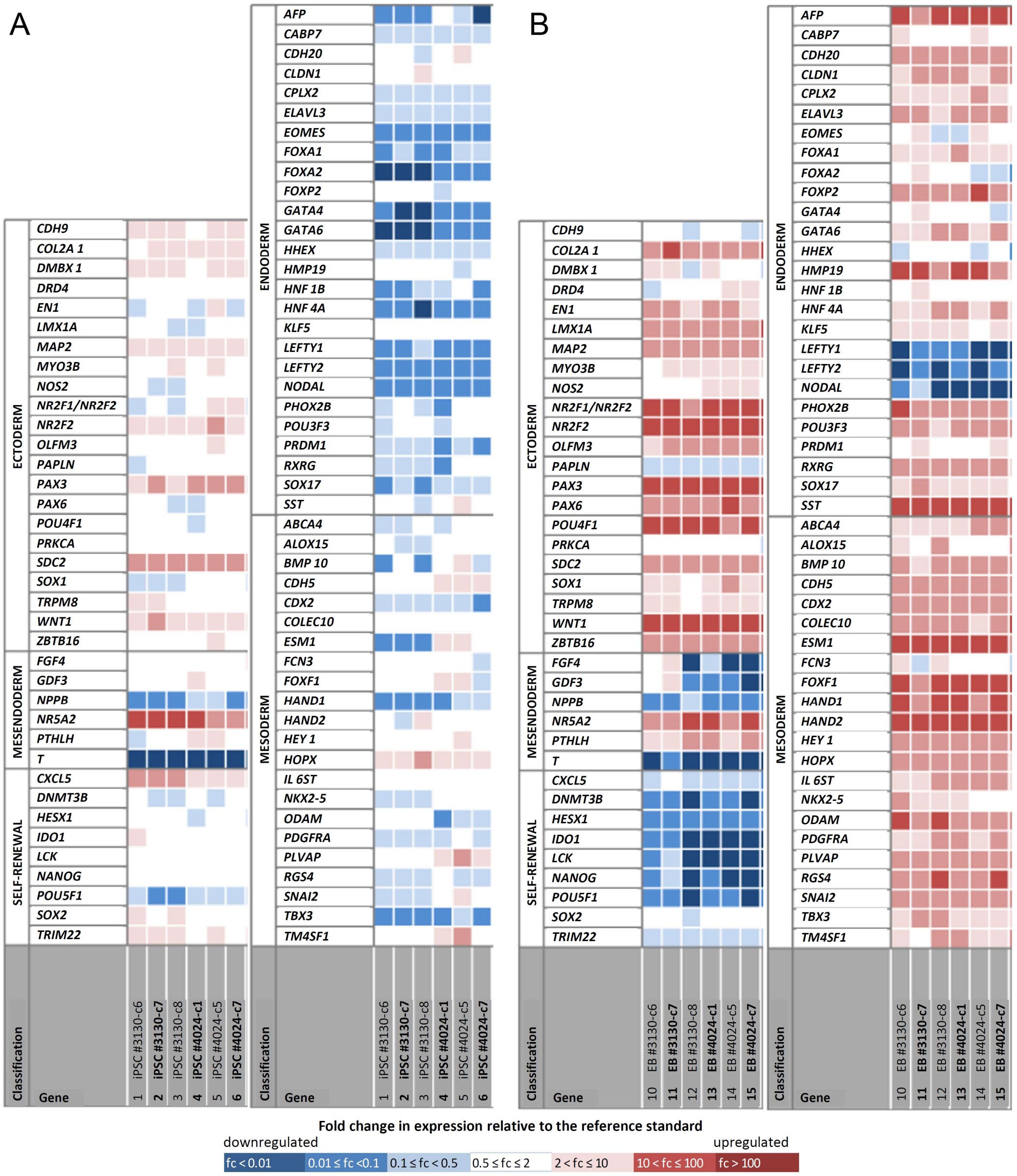

Figure S3. Scorecard analysis expression pattern plot for iPSCs (A) and EBs (B) relative to an undifferentiated reference set of nine undifferentiated pluripotent stem cell lines.

Table S1: List of RT-PCR primers used.

Target	5'-3' Forward primer	5'-3' Reverse primer	Amplicon length
SeV	GGATCACTAGGTGATATCGAGC*	ACCAGACAAGAGTTTAAGAGATATGTATC*	181 bp
KOS	ATGCACCGCTACGACGTGAGCGC	ACCTTGACAATCCTGATGTGG	528 bp
KLF4	TTCCTGCATGCCAGAGGAGCCC	AATGTATCGAAGGTGCTCAA*	410 bp
C-MYC	TAACTGACTAGCAGGCTTGTCG*	TCCACATACAGTCCTGGATGATGATG	532 bp
18S	GAGGATGAGGTGGAACCTGT	TCTTCAGTCGCTCCAGGTCT	166 bp
LIN28A	GTCTGGAATCCATCCGTGTC	GCTTCTGCATGCTCTTTCCT	87 bp
POU5F1	GAAGGATGTGGTCCGAGTGT	GCCTCAAAATCCTCTCGTTG	90 bp
RPE65	AAAGATCCCACCACTGCAAG	GATGGCTTGAATCGGTCACT	93 bp
BEST1	AAGACTGTGAGTTCTGGGGC	ACTCCACAGTTTTCCTCCTCAC	109 bp
MITF	CTCGAGCTCATGGACTTTCC	TGATGATCCGATTCACCAAA	72 bp
MCT3 splicing	TCATGCTAGCCATGCTCTACG	CAAAGCGGGTCACGAGGATG	687 bp

* Primer containing SeV genome sequences. Pairing of these primers with transgene-specific primers allows for the specific detection of transgenes carried by the vectors from the CytoTune® 2.0 Sendai reprogramming kit.

Table S2. List of antibodies used for immunocytochemistry analyses.

Antigen	Species	Dilution for immuno-fluorescence	Dilution for Western blotting	Reference
BESTROPHIN	Mouse monoclonal	1:100	1:1,000	Abcam, ab2182
EZRIN	Mouse monoclonal	1:250		Sigma, E8897
MCT1	Rabbit polyclonal	1:100	1:200	Millipore Chemicon, AB3538P
MCT3	Rabbit C-ter peptide	1:1,000	1:5,000	Nancy J. Philp
MITF	Mouse monoclonal	1:200		DAKO, M3621
NANOG	Rabbit monoclonal	1:200		Cell Signaling, #4903
OCT-4A	Rabbit monoclonal	1:150		Cell Signaling, #2840
RPE65	Rabbit monoclonal		1:1,000	Abcam, ab175936
SSEA-4	Mouse monoclonal	1:100		ThermoFischer Scientific, 41-4000
TRA1-81	Mouse monoclonal	1:100		ThermoFischer Scientific, 41-1100
α-TUBULIN	Mouse monoclonal		1:2,000	Sigma, T6199
ZO-1	Rabbit polyclonal	1:250		ThermoFischer Scientific, 61-7300
ZO-1	Mouse monoclonal	1:100		ThermoFisher Scientific, 33-9100
